# Impact of antigenic and genetic drift on the serologic surveillance of H5N2 avian influenza viruses

**DOI:** 10.1186/1746-6148-6-57

**Published:** 2010-12-20

**Authors:** Magdalena Escorcia, Karol Carrillo-Sánchez, Santiago March-Mifsut, Joaquin Chapa, Eduardo Lucio, Gerardo M Nava

**Affiliations:** 1 Departamento de Producción Animal Aves. Facultad de Medicina Veterinaria y Zootecnia. Universidad Nacional Autónoma de México. Ciudad Universitaria, Coyoacán, D. F., CP 04510, México; 2 Instituto Nacional de Medicina Genómica, Periférico Sur No. 4124, Torre Zafiro II, Piso 6. Col. Ex Rancho de Anzaldo, Alvaro Obregón. D. F. CP 01900. México; 3 Investigación Aplicada S. A. de C. V. 7 Norte 416. Tehuacán, Puebla. CP 75700. México; 4 Washington University School of Medicine. Dept. Pathology and Immunology. 660 S. Euclid Ave. St. Louis, MO 63110. USA

## Abstract

**Background:**

Serologic surveillance of Avian Influenza (AI) viruses is carried out by the hemagglutination inhibition (HI) test using reference reagents. This method is recommended by animal health organizations as a standard test to detect antigenic differences (subtypes) between circulating influenza virus, vaccine- and/or reference- strains. However, significant discrepancies between reference antisera and field isolates have been observed during serosurveillance of influenza A viruses in pig and poultry farms. The objective of this study was to examine the effects of influenza virus genetic and antigenic drift on serologic testing using standard HI assays and reference reagents. Low pathogenic AI H5N2 viruses isolated in Mexico between 1994 and 2008 were used for phylogenetic analysis of AI hemagglutinin genes and for serologic testing using antisera produced with year-specific AI virus isolates.

**Results:**

Phylogenetic analysis revealed significant divergence between early LPAI H5N2 viruses (1994 - 1998) and more recent virus field isolates (2002 - 2008). Results of the HI test were markedly influenced by the selection of the AI H5N2 virus (year of isolation) used as reference antigen for the assay. These analyses indicate that LPAI H5N2 viruses in Mexico are constantly undergoing genetic drift and that serosurveillance of AI viruses is significantly influenced by the antigen or antisera used for the HI test.

**Conclusions:**

Reference viral antigens and/or antisera need to be replaced constantly during surveillance of AI viruses to keep pace with the AI antigenic drift. This strategy should improve the estimation of antigenic differences between circulating AI viruses and the selection of suitable vaccine strains.

## Background

Avian Influenza (AI) virus belongs to the *Orthomyxoviridae *family, *Influenzavirus A *genus. This virus possesses eight segments of single-stranded RNA genome. Two of these segments encode for two important membrane glycoproteins, hemagglutinin (HA) and neuraminidase (NA) [1], that play a key role during cellular infection. These two proteins are used for virus subtype classification [1,2]. Also, depending on severity of disease in avian species, AI viruses are categorized into highly- and low-pathogenic (HPAI and LPAI, respectively) viruses [1,2].

In response to the recent cases of human infections caused by HPAI H5N1 viruses, authorities and scientists were encouraged to review and apply policies for effective surveillance and control of AI infections [3-5]. In many countries, the use of AI vaccines was banned or discouraged because vaccination programs could interfere with appropriate detection of HPAI outbreaks [5]. However, the use of AI vaccines has been reconsidered by some countries due to the recent increase in AI cases in commercial farms and devastating consequences for human health [5].

In Mexico, an AI vaccination program was established in 1994. Initially, the program was instituted to control the HPAI H5N2 virus outbreak that occurred during that year [6]. A commercial vaccine against AI was produced using the officially authorized virus strain A/Ck/México/CPA-232/1994(H5N2). A few months later, the HPAI virus was eradicated from Mexico and it was decided to continue the vaccination program to protect commercial flocks from LPAI H5N2 viruses [6].

After almost two decades of using the AI vaccine in Mexico, commercial farms remain HPAI-free. However, veterinary services have observed an increase in respiratory signs in vaccinated, field challenged (LPAI virus) birds. Moreover, animal health laboratories have reported significant differences in the hemagglutination inhibition (HI) tests between field LPAI H5N2 isolates and the vaccine strain [7]. These discrepancies observed during AI surveillance could be attributed to a gradual accumulation of antigenic drift. In fact, it was shown that LPAI H5N2 viruses in Mexico are constantly undergoing genetic drift, and that recent AI virus isolates have significant antigen divergence when compared to the AI vaccine strain [7].

In Mexico, as in many other countries, AI surveillance is primarily carried out by the HI test using reference antigens or antisera [8,9]. This method is recommended by the World Organization for Animal Health (OIE) as standard test to detect antigenic differences (subtypes) between circulating, vaccine and reference AI virus strains [8,10], and to evaluate vaccine efficacy [8,11,12]. Antigens for production of vaccines or antisera are maintained and distributed by official reference laboratories [10] and in many cases, these antigens are produced with AI viruses isolated more than a decade ago (e.g. [13,14]).

Although the HI test and reference antigens are used worldwide for AI surveillance; little has been done to examine the effects of AI antigenic drift on the antigenic surveillance of field strains. We hypothesize that the antigenic drift that occurred in recent field isolates of H5N2 virus produced significant variation in the accuracy of the serologic surveillance. The objective of the present study was to examine the effects of AI antigenic drift on immune reactivity of reference antisera using standard HI tests.

## Results

Low pathogenic AI H5N2 viruses isolated in Mexico between 2002 and 2008 were used for phylogenetic analyses of AI hemagglutinin genes and immune reactivity using antisera produced with AI virus isolated in different years.

### Phylogenetic analysis of Avian Influenza hemagglutinin genes

Field isolates of LPAI H5N2 virus were used for this study. These viruses were isolated from vaccinated birds that developed the clinical presentation of the disease. Virus were replicated in chicken embryo and reverse transcriptase PCR was used for the amplification of the HA gene (between nucleotide positions 451 and 1262), a marker for the virulence potential of AI viruses [15]. HA gene segments were sequenced and annotated for phylogenetic analyses. To accomplish a more comprehensive evolutionary history of AI H5N2 viruses in Mexico, all available A/Ck/México/H5N2 sequences in the Influenza Virus Resource at the National Center for Biotechnology Information [16] were retrieved and used for genetic analysis.

**Figure 1:**
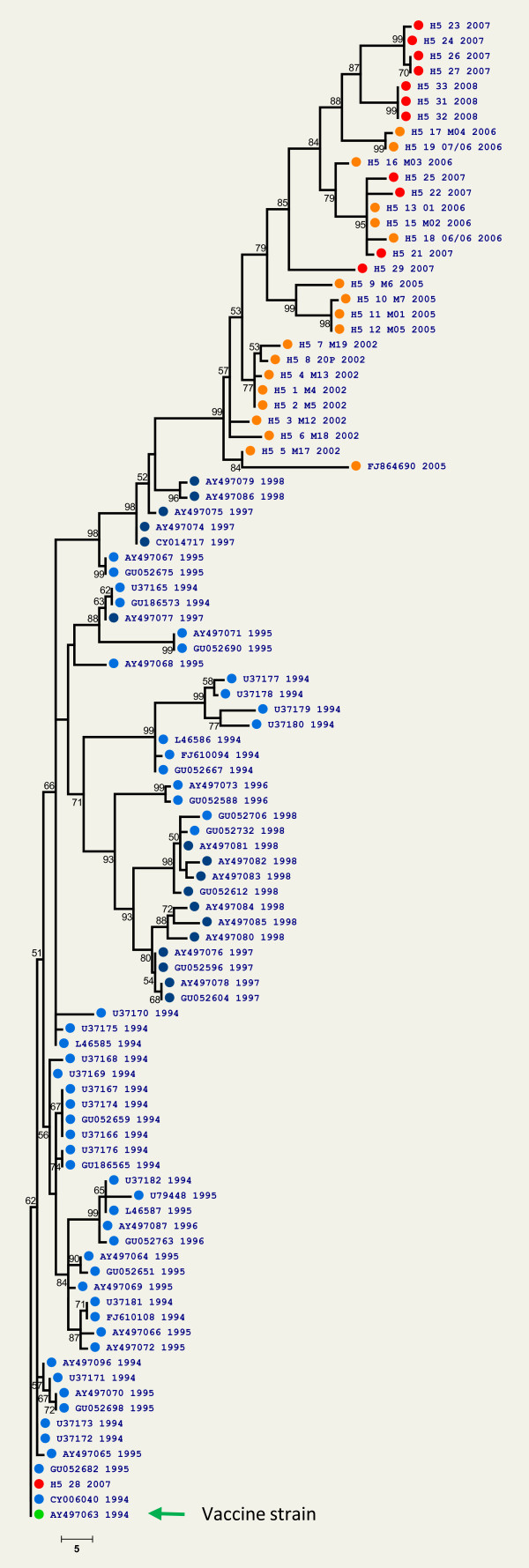
**Phylogenetic rooted tree based on partial nucleotide sequences (≈812 pb**.) of the hemagglutinin (HA) gene from low pathogenic Avian Influenza H5N2 viruses isolated between 2002 and 2008. Reverse transcriptase PCR was used for the amplification of the HA cleavage site sequence of different Avian Influenza viruses isolated in Mexico. Maximum parsimony and best heuristic tree search analysis showing the relationships of nucleotide sequences of HA genes. Similar tree topologies were obtained by the maximum likelihood method. Tree was rooted using the nucleotide sequence from the vaccine strain (AY497063). Numbers on branches indicate bootstrap values after 1,000 replicates. Scale bar indicates the number of changes over the whole sequence. Low pathogenic Avian Influenza H5N2 viruses isolated in Mexico between 1994-1996 (light-blue circles), 1997-1998 (dark-blue circles), 2002-2006 (orange circles) and 2007-2008 (red circles).

Phylogenic analysis of partial HA gene sequences from AI H5N2 viruses isolated between 1994 and 2008 revealed that the AI viruses are constantly undergoing genetic drifts. Phylogenetic trees derived from partial nucleotide sequences demonstrated that HA genes amplified from field LPAI H5N2 isolates distinctively cluster by year of isolation (Figure 1). Similar tree topologies were obtained by the maximum likelihood method (data not shown). These phylogenetic analysis were confirmed by estimating percent sequence identity scores for pairwise comparisons of LPAI H5N2 isolates and the vaccine strain (Table 1). Together, these data indicate that recent LPAI H5N2 isolates (2007 - 2008) have undergone significant molecular drifts when compared to the vaccine strain (A/Ck/México/CPA-232/1994), isolated in 1994 and early viruses isolated between 1994 and 1998 (Figure 1 and Table 1). This analysis confirms that HA gene lineages in Mexico follow a yearly cumulative trend of sequence mutations. Interestingly, one of the most recent virus isolates (sample ID: H5 28 2007) isolated in 2007, was closely related to the vaccine strain and early virus isolates (Figure 1). This result could indicate that at some point a vaccine-like virus may have circulated in commercial flocks during 2007.

**Table 1:** Percent of sequence similarity between the reference strain and field isolates

Year of isolation	Percentage of sequence identity
1994 (n = 29)	97.7 ± 2.1^a^
1995 (n = 16)	97.5 ± 1.4^a^
1996 (n = 4)	96.5 ± 0.5^ab^
1997 (n = 8)	96.0 ± 0.5^b^
1998 (n = 11)	94.5 ± 0.5^b^
2002 (n = 8)	92.7 ± 0.4^c^
2005 (n = 5)	90.8 ± 0.4^d^
2006 (n = 6)	90.1 ± 0.4^d^
2007 (n = 9)	91.1 ± 3.3^c^
2008 (n = 3)	91.0 ± 0.0^c^

Percent of sequence similarity between the reference strain (A/Ck/México/1994) and low pathogenic Avian Influenza H5N2 viruses isolated between 1994 and 2008. Mean values ± SD. Statistical comparisons were made using ANOVA and Fisher's protected least significant difference test. Values with different superscript are significantly different (*P *≤ 0.05).

The phylogenetic analysis also revealed significant divergence between different strains of LPAI H5N2 isolates in Mexico. Viruses circulating between 1994 and 1998 were highly divergent to viruses isolated between 2002 and 2008 (Figure 1 and Table 1). These results indicate that current AI virus circulating in Mexico possesses sufficient genetic divergence to warrant a vaccine strain update. To assess the effect of this antigenic divergence between field isolates and the official vaccine strain, serologic testing was evaluated by means of HI assays.

### Serologic testing using hemagglutination inhibition tests

The thirty-four field isolates of LPAI H5N2 virus were used to evaluate immune reactivity using standard HI tests. AI antisera was produced using seven year-specific AI H5N2 antigens: A) A/Ck/México/CPA-232/1994, reference antigen and virus strain used for vaccine production; B) A/Ck/México/2002; C) A/Ck/México/2003; D) A/Ck/México/2005; E) A/Ck/México/2006; F) A/Ck/México/2007 and G) A/Ck/México/2008. The HI assay demonstrated highly variable results when field LPAI H5N2 viruses where tested against antisera derived from year-specific AI H5N2 strains.

In the HI tests using antiserum produced with antigen A/Ck/México/CPA-232/1994, geometric mean titers (GMT) were significantly higher in LPAI H5N2 viruses isolated in 2002 compared to viruses isolated between 2006, 2007 and 2008 (Table 2). The HI test GMT of viruses isolated between 2002 and 2005 were comparable. These results indicate that at the HA antigenic level, 2002 and 2005 LPAI viruses were more closely related to A/Ck/México/CPA-232/1994 than more recent virus isolates (2006 - 2008) (Table 2).

**Table 2:** Antigenic relatedness of the reference strain and field isolates

HI test antigen	Antisera						
	México/1994 (A)	México/2002 (B)	México/2003 (C)	México/2005 (D)	México/2006 (E)	México/2007 (F)	México/2008 (G)
2002 (n = 8 isolates)	135^a^	987^a^	287	207	538^a^	494^b^	87^b^
2005 (n = 5 isolates)	70^ab^	211^b^	160	279	320^a^	485^b^	92^b^
2006 (n = 6 isolates)	32^b^	905^a^	113	254	718^a^	1810^a^	285^ab^
2007 (n = 10 isolates)	37^b^	1114^a^	260	171	1040^a^	1940^a^	485^a^
2008 (n = 5 isolates)	80^b^	160^b^	160	23	70^b^	160^b^	422^ab^

Antigenic relatedness of the reference strain (A/Ck/México/1994) and low pathogenic Avian Influenza H5N2 viruses isolated between 2002 and 2008 tested by hemagglutination inhibition (HI) test. Official virus ID: A). A/Ck/México/1994; B). A/Ck/México/2002; C). A/Ck/México/2003; D). A/Ck/México/2005; E). A/Ck/México/2006; F). A/Ck/México/2007; G). A/Ck/México/2008. Geometric mean values for HI test using different antisera (A-G) and low pathogenic Avian Influenza H5N2 viruses isolated between 2002 and 2008. Statistical comparisons were made using ANOVA and Fisher's protected least significant difference test. Columns with different superscript are significantly different (*P *≤ 0.05).

For the HI test using antiserum produced with antigen A/Ck/México/2002, GMT revealed close homology between the 2002 virus isolates; however, these GMT did not follow a yearly trend for the other years. For example, 2005 LPAI isolates were antigenically divergent to A/Ck/México/2002 whereas no differences were detected between viruses isolated in 2006 and 2007. In contrast, 2008 isolates were antigenically divergent when compared to 2007 viruses; indicating highly antigenic divergence between viruses isolated at close time points (Table 2).

When A/Ck/México/2003 and A/Ck/México/2005 antisera were used for the HI assays, antigenic homology was comparable between LPAI viruses isolated in 2002, 2005, 2006, 2007 and 2008. These results indicate that at the HA antigenic level, this collection of LPAI viruses possess comparable antigenic homology with the A/Ck/México/2003 virus (Table 2). Nevertheless, it is noteworthy that the GMT in the HI test were highly variable.

In the HI test using the A/Ck/México/2006, LPAI viruses isolated between 2002 and 2007 showed comparable antigenic homology. In contrast, viruses isolated during 2008 showed a reduced antigenic homology when compared to 2007 LPAI isolates. (Table 2).

**Figure 2:**
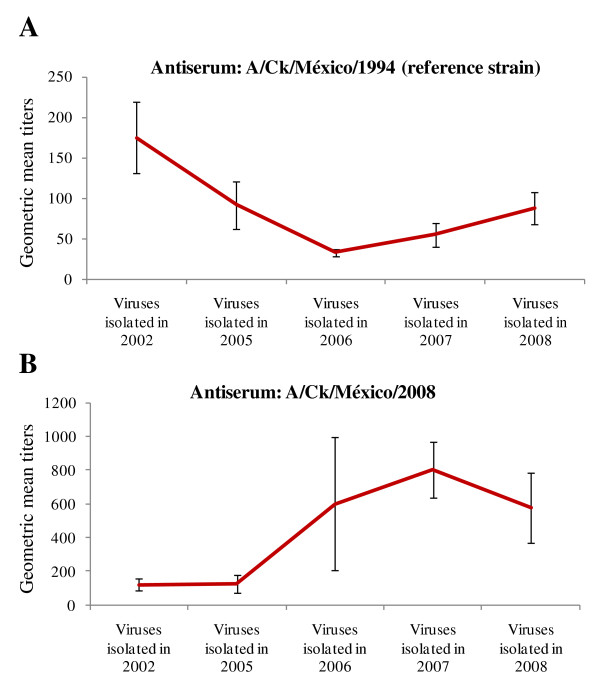
**Examples of yearly trends in hemagglutination inhibition (HI) titers of low pathogenic Avian Influenza H5N2 viruses isolated in Mexico between 2002 and 2008**. This figure depict results of the HI test using antiserum produced with the reference antigen A/Ck/México/CPA-232/1994 or antiserum produced with a recent field isolate A/Ck/México/2008. Geometric mean values ± SE are presented. The complete dataset is included in Table 2.

The HI test using antiserum A/Ck/México/2007 followed a yearly trend. LPAI viruses isolated in 2006 and 2007 showed comparable antigenic homology. In contrast, these LPAI isolates were antigenically divergent to early isolates (2002 and 2005) and more recent isolates (2008). These results indicate the LPAI H5N2 viruses circulating during 2006 and 2007 possess year-specific antigenic divergence compared to other years of isolation. Comparable trend was observed in the HI test using the A/Ck/México/2008 antiserum. LPAI viruses circulating between 2006 and 2007 were more antigenically related to the A/Ck/México/2008 virus (Table 2). These results corroborate that recent AI viruses have accumulated significant antigenic drifts to be distinguishable from early AI isolates (e.g. 2002 and 2005). Taken together, these results show some yearly tendencies in antigenic homology in which early LPAI isolated strains (e.g. 2002) are more antigenically related to the vaccine strain A/Ck/México/CPA-232/1994 and recent LPAI isolates (2007 and 2008) more antigenically related to the A/Ck/México/2008 virus (Figure 2). These results indicate that the antigenic differences of circulating LPAI H5N2 viruses may warrant a new vaccine and serosurveillance antigen update.

## Discussion

Worldwide, human and animal health organizations have established detailed schemes for influenza surveillance. These programs rely on serological assays to characterize virus subtypes, establishing seroprevalence and evaluating vaccine efficacy [8,11,17]. In veterinary medicine, the HI test is the standard technique used to detect antigenic differences between circulating influenza virus, vaccine- and/or reference- strains [8,9,17,18].

Because the HI test is used worldwide for AI surveillance, it is essential to identify potential pitfalls of using this serologic test. The objective of the present study was to examine the effects of AI antigenic drift on serosurveillance using reference antisera during standard HI tests. We amplified, sequenced and analyzed partial AI hemagglutinin genes and performed serologic tests using LPAI H5N2 viruses isolated in Mexico between 1994 and 2008. Phylogenetic analyses revealed that molecular drift in HA gene follow a yearly trend, suggesting gradually cumulative sequence mutations. Recent (2006 - 2008) field isolates of LPAI H5N2 viruses in Mexico have undergone important antigenic drift in the HA gene when compared to early LPAI isolates or vaccine strains (1994 - 1996). Viruses isolated between 1994 and 1998 cluster in distinctive and divergent lineages compared to viruses isolated between 2002 and 2008. Comparable evolutionary trends were observed in previous analyses of LPAI H5N2 lineages in Mexico [7,19].

These evolving genetic drifts observed in HA genes of LPAI H5N2 may explain the increasing incidence of respiratory signs in vaccinated, field challenged birds, and the discrepancies observed during LPAI H5N2 serosurveillance. To confirm this idea, serologic testing was carried out using an array of seven different reference AI antisera produced with LPAI H5N2 antigens isolated in 1994, 2002, 2003, 2005, 2006, 2007 and 2008. These analyses revealed that HI test results are significantly influenced by the selection of year-specific antigens. Generally, early LPAI H5N2 virus isolates (e.g. 2002) produced higher HI titers with antisera produced with early isolates (e.g. 1994). In contrast, more recent LPAI H5N2 isolates (i.e. 2007 and 2008) produced higher HI titers with antisera produced with recent LPAI isolated strains (i.e. year 2008). These results explain the high variability of LPAI titers in commercial flocks when the reference AI antigen A/Ck/México/CPA-232/1994(H5N2) is used in the HI test.

Despite that considerable efforts have been made to produce reference antibodies to improve the accuracy of the HI test during serosurveillance of AI [20,21], the accelerated mutation rate of AI viruses and the rapid accumulation of antigen drift [7,19,22] require a recurrent update of reference antigens or antisera for accurate surveillance. For example, in Mexico around 2004, veterinary services reported that AI serum titers in commercial flocks were highly variable when the reference antigen A/Ck/México/CPA-232/1994(H5N2) (isolated in 1994) was used for the HI test. Similar discrepancies between reference antisera and field isolates were observed in the serosurveillance of H3N2 and H1N1 influenza A viruses in pigs [23,24].

Our results confirm the idea that the accelerated antigenic drift observed in AI viruses, not only affects the performance and accuracy of the HI test during AI serosurveillance but also, the effectiveness of AI vaccines [7,19,22]. Hence, for an effective control of AI H5N2 viruses, reference antigens and vaccine strains must be replaced constantly to keep pace with the AI antigenic drift. This approach has been a successful strategy for the control and surveillance of human influenza [25].

## Conclusions

The present study reveals that due to the rapid antigenic drift of AI viruses, standardization and constant renewal of reference antigens is required during the establishment of influenza serosurveillance programs. More importantly, these data provide clear evidence of the impact of antigenic drifts on the evasion of the immune system. Antibodies produced against early AI viruses (e.g. A/Ck/México/CPA-232/1994) have reduced effectiveness against LPAI viruses currently circulating in the environment. Thus, the observed genetic and antigenic differences of circulating LPAI H5N2 viruses warrant an update of the vaccine strain and serosurveillance antigens.

## Methods

### Avian Influenza field isolates

Thirty-four field isolates of LPAI virus, subtype H5N2 were used for the study. These viruses were isolated in 2002, 2005, 2006, 2007 and 2008 from vaccinated birds that developed the clinical presentation of the disease. These isolates were previously reported to the Mexican Ministry of Agriculture, Livestock, Rural Development, Fisheries and Food (SAGARPA, its Spanish acronym).

### Hemagglutination inhibition assays

The thirty-four field isolates of LPAI H5N2 virus were evaluated by the standard HI test [12] using chicken antisera produced with seven different AI H5N2 antigens: A) A/Ck/México/CPA-232/1994, reference antigen and virus strain used for vaccine production; B) A/Ck/México/2002; C) A/Ck/México/2003; D) A/Ck/México/2005; E) A/Ck/México/2006; F) A/Ck/México/2007 and G) A/Ck/México/2008. Reference antisera were prepared by conventional methods using inactivated viruses [26].

### Amplification of Avian Influenza hemagglutinin gene

Viral RNA extraction from allantoic fluid was performed using conventional methods [27]. Reverse transcriptase PCR (RT-PCR) was used for the amplification of the HA cleavage site sequence (from positions 451 to 1262 of the HA gene using sequence A/turkey/Ontario/7732/66 as numbering system; accession number AB558456), a marker for the virulence potential of AI viruses [15]. PCR protocols and primer sequences are described elsewhere [15]. Amplifications of an 812 bp. HA gene segment were performed using a RT-PCR kit (SuperScript One-Step RT-PCR with Platinum Taq; Carlsbad, CA). After visual confirmation of the PCR products with agarose gel electrophoresis, PCR products were purified using the QIAquick PCR Purification Kit (Qiagen, Valencia, CA). Sequencing of the HA gene segments was performed using the 3730XL automated sequencer (Applied Biosystems, CA. USA).

### Phylogenetic analysis of Avian Influenza hemagglutinin genes

Nucleotide sequences obtained from the LPAI H5N2 field isolates were inspected, trimmed and assembled using the Sequencher 4.9 software (Ann Arbor, MI). To perform a comprehensive evolutionary analysis of AI H5N2 viruses in Mexico, all available H5N2 sequences were also retrieved from the Influenza Virus Resource at the National Center for Biotechnology Information [16]. Nucleotide sequences were aligned using ClustalW software [28], manually inspected for quality and trimmed to equal length. Aligned sequences were used for phylogenetic analysis using the maximum parsimony and maximum likelihood methods [29]. Analysis were performed using the MEGA4 [30], PhyML [31] and Seaview [32] software. Trees were rooted using [GenBank accession number AY497063] (vaccine strain) nucleotide sequence. The statistical significance of branch order was estimated by the generation of 1000 replications of bootstrap re-sampling of the originally-aligned nucleotide sequences.

### Statistical analysis

Results of HI test assays for each LPAI H5N2 virus were used to compare GMT between different year-specific antisera. Analyses were performed using SAS software (Statview, Version 5.0.1; SAS Institute, Cary, NC). ANOVA and Fisher's protected least significant difference test. Results were considered significant if P ≤ 0.05.

### Nucleotide sequence accession numbers

The sequences of the HA genes identified in this work are deposited in GenBank under accession numbers: HM998867 through HM998896.

## List of Abbreviations

AI: avian influenza; ANOVA: analysis of variance; HA: Hemagglutinin; HI: hemagglutination inhibition; HPAI: highly pathogenic avian influenza; LPAI: low pathogenic avian influenza; NA: neuraminidase; RT-PCR: reverse transcriptase polymerase chain reaction; SAGARPA: Ministry of Agriculture; Livestock; Rural Development; Fisheries and Alimentation (SAGARPA, its Spanish acronym).

## Competing interests

The authors declare that they have no competing interests.

## Authors' contributions

ME, EL and GMN designed experiments; ME, KCS, SMM, and JC performed research; GMN and ME analyzed data; GMN and ME wrote the paper. All coauthors read and approved the final manuscript.
